# Characterization of clinical data for patient stratification in moderate osteoarthritis with support vector machines, regulatory network models, and verification against osteoarthritis Initiative data

**DOI:** 10.1038/s41598-024-62212-x

**Published:** 2024-05-23

**Authors:** Maria Segarra-Queralt, Mar Galofré, Laura Tio, Jordi Monfort, Joan Carlos Monllau, Gemma Piella, Jérôme Noailly

**Affiliations:** 1 BCN MedTech, Department of Engineering, Universitat Pompeu Fabra, 08018 Barcelona, Spain; 2 IMIM (Hospital del Mar Medical Research Institute), Hospital del Mar, 08003 Barcelona, Spain; 3 Rheumatology Department, Hospital del Mar, 08003 Barcelona, Spain; 4 Orthopedic Surgery and Traumatology Department, Hospital del Mar, 08003 Barcelona, Spain

**Keywords:** Biomarkers, Molecular medicine, Rheumatology, Biochemical networks, Computational science

## Abstract

Knee osteoarthritis (OA) diagnosis is based on symptoms, assessed through questionnaires such as the WOMAC. However, the inconsistency of pain recording and the discrepancy between joint phenotype and symptoms highlight the need for objective biomarkers in knee OA diagnosis. To this end, we study relationships among clinical and molecular data in a cohort of women (n = 51) with Kellgren–Lawrence grade 2–3 knee OA through a Support Vector Machine (SVM) and a regulation network model. Clinical descriptors (i.e., pain catastrophism, depression, functionality, joint pain, rigidity, sensitization and synovitis) are used to classify patients. A Youden’s test is performed for each classifier to determine optimal binarization thresholds for the descriptors. Thresholds are tested against patient stratification according to baseline WOMAC data from the Osteoarthritis Initiative, and the mean accuracy is 0.97. For our cohort, the data used as SVM inputs are knee OA descriptors, synovial fluid proteomic measurements (n = 25), and transcription factor activation obtained from regulatory network model stimulated with the synovial fluid measurements. The relative weights after classification reflect input importance. The performance of each classifier is evaluated through ROC-AUC analysis. The best classifier with clinical data is pain catastrophism (AUC = 0.9), highly influenced by funcionality and pain sensetization, suggesting that kinesophobia is involved in pain perception. With synovial fluid proteins used as input, leptin strongly influences every classifier, suggesting the importance of low-grade inflammation. When transcription factors are used, the mean AUC is limited to 0.608, which can be related to the pleomorphic behaviour of osteoarthritic chondrocytes. Nevertheless, funcionality has an AUC of 0.7 with a decisive importance of FOXO downregulation. Though larger and longitudinal cohorts are needed, this unique combination of SVM and regulatory network model shall help to stratify knee OA patients more objectively.

## Introduction

Synovial joints (e.g., knee, hip and hand) allow smooth movements between adjacent bones and are surrounded by an articular capsule that defines a cavity filled with synovial fluid. Bone extremities are also covered by a layer of hyaline articular cartilage that prevents bone-to-bone contact, cushions possible noxious impacts, and provides a low-friction surface for joint articulation^1^. Epidemiological data from the Institute for Health Metrics and Evaluation shows that knee osteoarthritis (OA) affects 22% of men and 31% of women over the age of 55 in 2019. Such prevalence is expected to rise due to the increase in life expectancy and the overall body mass index. Currently, treatments are conservative (i.e., weight loss, low-impact exercises and analgesics) and knee OA-modifying drugs are not available because knee OA pathophysiology is not fully understood. Eventually, total knee replacement may be necessary. Predictions suggest that the need for joint replacement will continue to rise because of both changes in patients’ demography, and expanding indications for surgery^2^. Consequently, the medical costs associated with knee OA are increasing, making this disease one of the world’s leading health problems^3^.

knee OA diagnosis occurs mainly during its moderate to severe/late stage when the articular cartilage has become irreversibly damaged. At this point, the decision to propose the patient a total knee replacement or conservative treatment considers the patient’s age and the extension of joint structural alterations, but the eventual decision is largely conditioned by the symptoms, pain being the critical one. Every person has a unique perception, as it is affected by biological, psychological and social factors^4^. Pain is typically assessed through questionnaires such as Western Ontario and McMaster Universities Osteoarthritis Index (WOMAC). However, the inconsistency and poor reporting of questionnaires, along with a poor consistency between physical joint damage, as assessed through radiological biomarkers, and pain symptoms, motivate the search for more objective measures that could be used as new biomarkers in knee OA diagnosis^5,6^.

To find such biomarkers, gait data have been used to develop prediction models for the progression of knee OA^7^. Also, serum and urine biochemical markers have been explored to establish knee OA phenotype classification systems: urinary C-terminal crosslinked telopeptide of type II collagen and serum degrading enzymes levels, cartilage oligomeric matrix protein and hyaluronan have some predictive value for knee OA classification^5^. But, as pain, at least the nociceptive dimension might be correlated with the levels of synovial inflammation^8^, there is a growing interest in exploring synovial fluid molecules as possible biomarkers^9,10^. For example, Haraden et al.^11^ predicted the development of inflammatory knee OA endotype based on synovial fluid molecules^11^. Synovial fluid data have also been combined with gait data to enable the construction of support vector machine classifiers to predict cartilage damage by Donnenfield et al.^10^.

But, patient stratification systems that helped to differentiate between sensory and affective pain dimensions from the nociceptive extent have not been fully explored^5^. Therefore, objectively and biologically characterizing current knee OA clinical descriptors (i.e., WOMAC domains, catastrophism, or sensitization) may help to gain valuable insight into patient stratification which eventually might help in clinical decision-making. Machine learning models can link seemingly unrelated features by finding patterns in training data. Specifically, this cross-sectional study aims to describe a cohort of women (n = 51) by mining the relationships among typical knee OA descriptors and molecular data through Support Vector Machine (SVM) classifiers to provide objectivity in current knee OA diagnostic systems. We further intend to add interpretability to the results by enriching proteomic real-world data. Specifically, by using as initial conditions the proteomic data we personalized simulations of a previously developed regulation network model^12^. The transcription factor results out of the regulatory model were used as the third set of input features for the SVM, allowing us to characterize the cohort with intracellular molecular information.

## Results

We explore a total of 686 classification tasks to classify a cohort of 51 women regarding individual levels of functionality (FU), rigidity (RI), joint pain (JP), pain catastrophism (CA), pain sensitization (SE), depression (DE) and synovitis (SY). Three different types of datasets are used as input features: (i) sets of knee OA-clinical features; (ii) synovial proteomics data; (iii) in-silico data regarding the activation of transcription factors from a regulatory network-based model.

**Figure 1:**
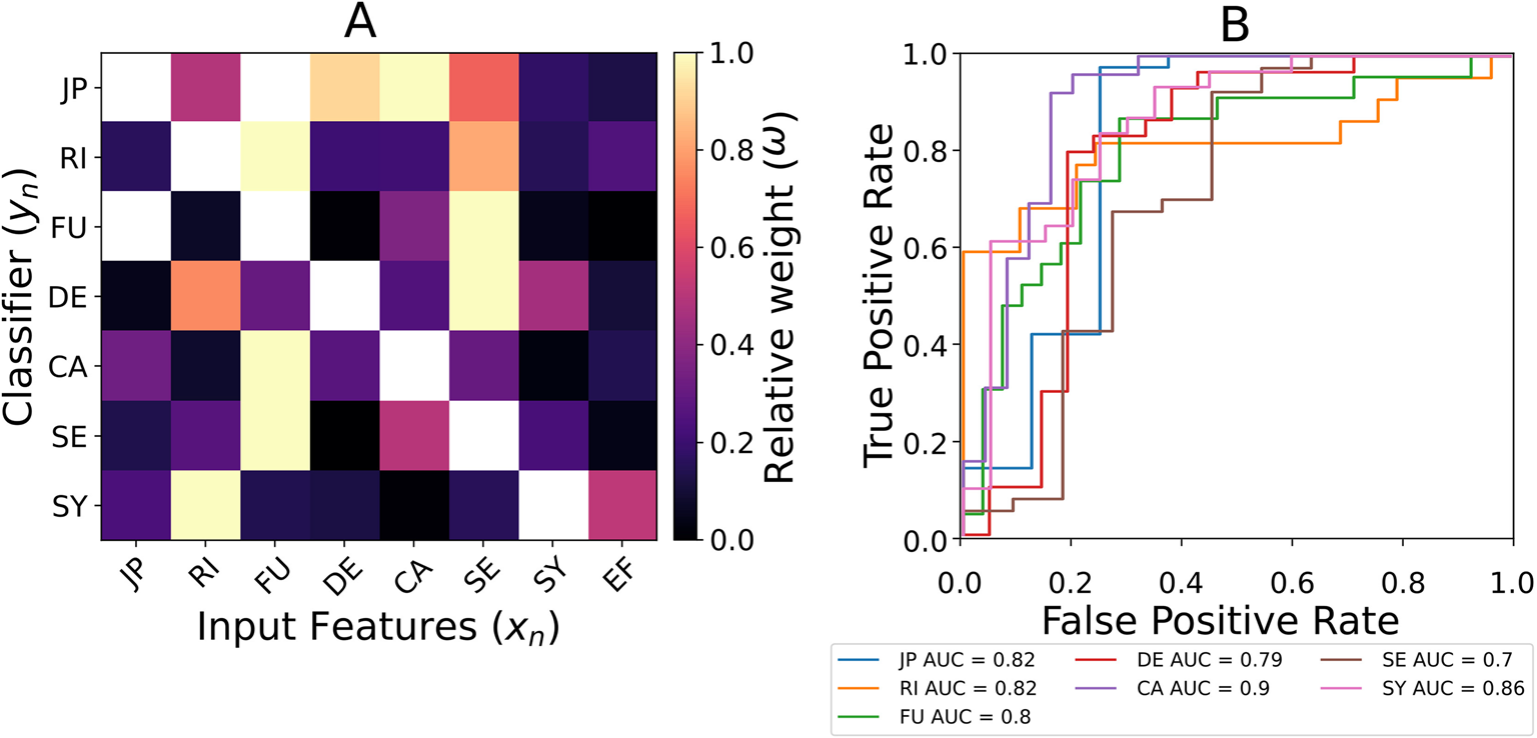
(**A**) Normalized importance when classifying each knee OA-descriptor with specific sets of knee OA-descriptors as input features (the omitted features for each classifier are depicted in white). (**B**) ROC-AUC analysis for the classifiers explored in A. functionality (FU), rigidity (RI), joint pain (JP), pain catastrophism (CA), pain sensetization (SE), depression (DE) and synovitis (SY).

Figure 1A shows the relative importance of each clinical variable when target knee OA descriptors (those that can be binarized with an $accuracy > 0.75$ on the train set) are classified: the closer the weight is to 1 (or yellow according to the colour bar employed), the more relevant such feature is for the classification. WOMAC domains classification (i.e., JP, RI and FU) are more influenced by CA, FU and SE, respectively ($AUC > 0.8$). DE is mostly influenced by FU, but in this case, the AUC score is lower ($AUC = 0.79$). CA classifier has the highest AUC score ($AUC = 0.9$) and is highly influenced by FU. The inflammatory classifier (SY) is mostly influenced by RI.

**Figure 2:**
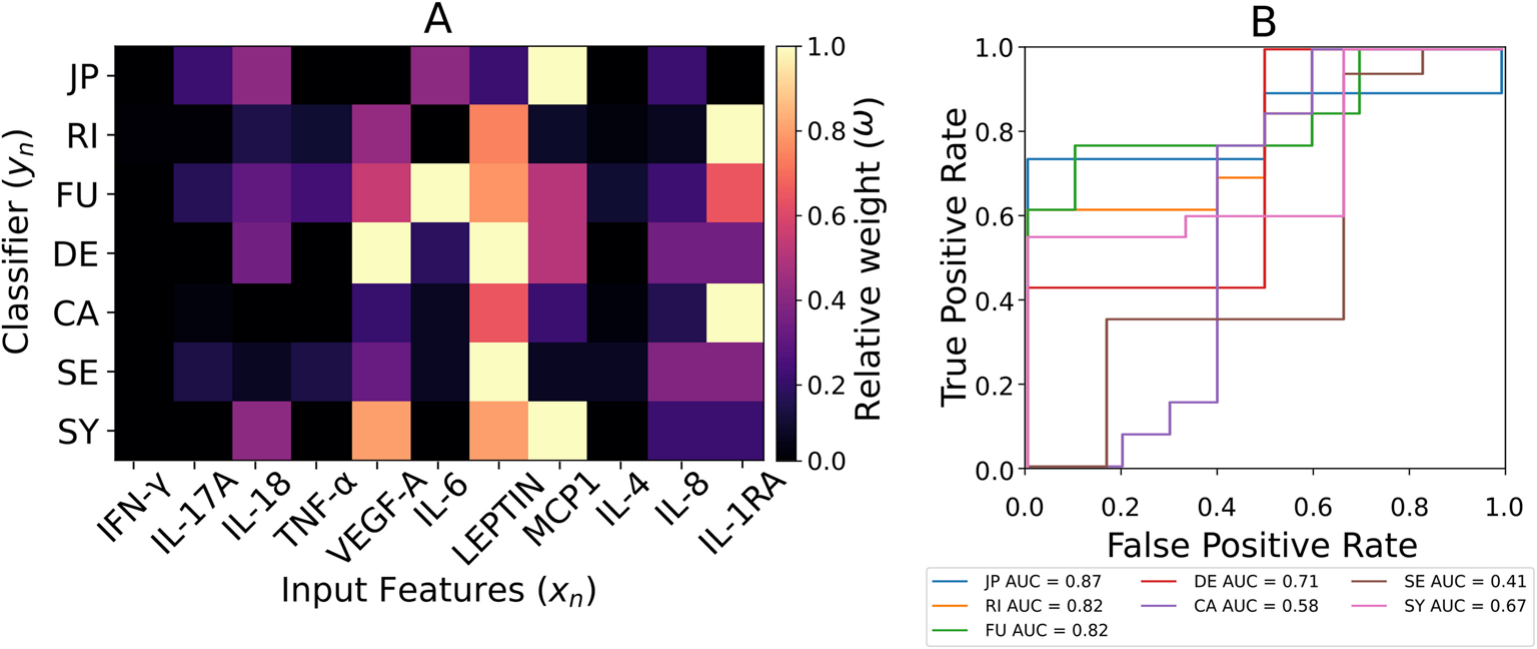
(**A**) Normalized importance when classifying knee OA-descriptors with synovial proteomics data as input features. (**B**) ROC-AUC analysis for the classifiers explored in A. functionality (FU), rigidity (RI), joint pain (JP), pain catastrophism (CA), pain sensetization (SE), depression (DE) and synovitis (SY).

Figure 2A shows the relative importance of SF molecules to classify knee OA clinical features. The classification of WOMAC domains (i.e., JP, RI and FU) are highly influenced ($AUC > 0.8$) by MCP1, IL-1RA and IL-6, respectively. With a lower score ($AUC = 0.71$), DE categorization is equally influenced by LEPTIN and VEGF-A. When classifying patients against CA and SE, IL-RA and LEPTIN are the parameters that have a higher influence, but the AUC score decreases below 0.6, reaching 0.41 for the SE classifier. SY is relatively well classified ($AUC = 0.67$), but in this case, the most influencing parameter is MCP1.

**Figure 3:**
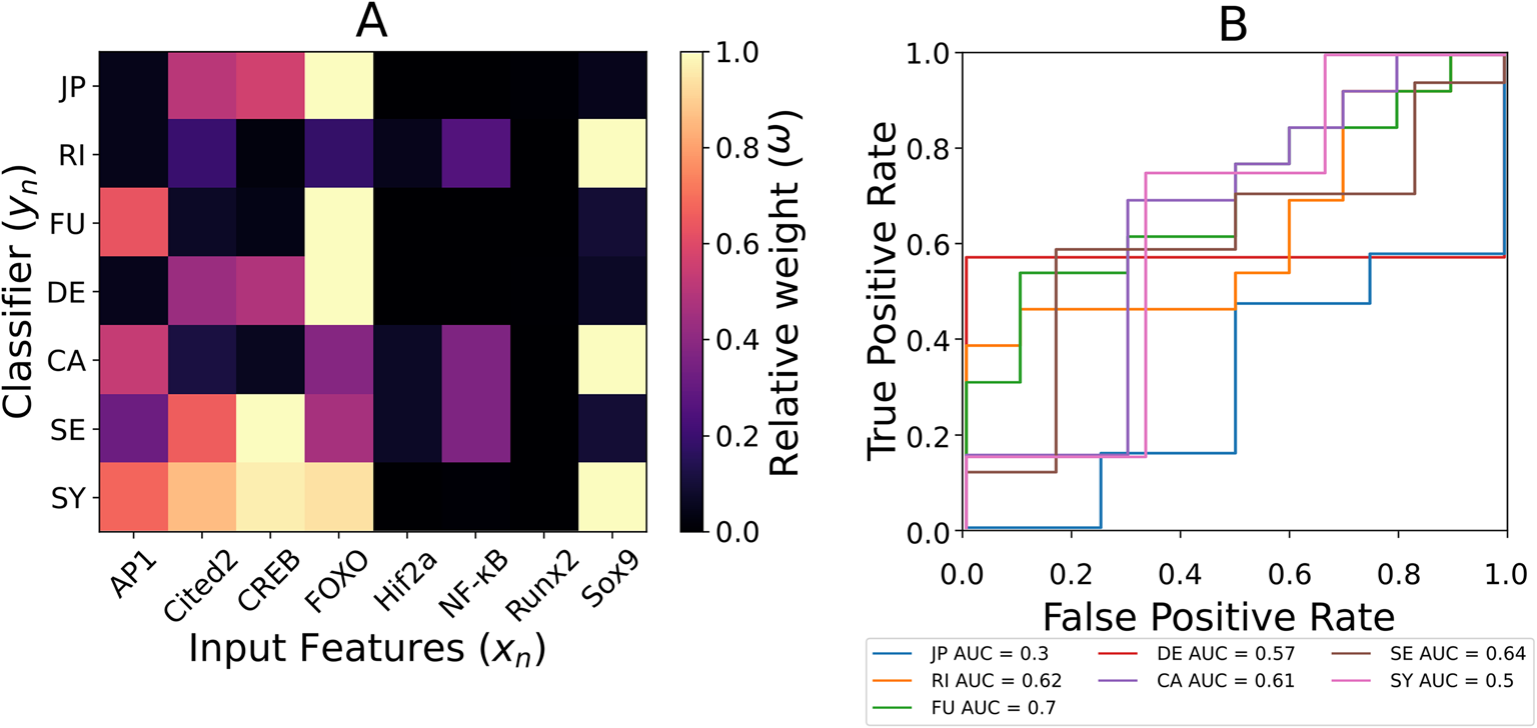
(**A**) Normalized importance when classifying knee OA-descriptors with transcription factor activities simulated with. a regulatory network-based model, as input features. (**B**) ROC-AUC analysis. functionality (FU), rigidity (RI), joint pain (JP), pain catastrophism (CA), pain sensetization (SE), depression (DE) and synovitis (SY).

Figure 3 shows the classification of knee OA clinical descriptors for n = 25 when the simulated activities of the TF of the regulatory network-based model of a chondrocyte^12^ are used as input features. Generally FOXO and Sox9 are the most influential TF (see Fig. 3A). Figure 3B reveals the robust effect of FOXO in JP and FU classification as it discriminates either the false positives ($AUC = 0.3$) or the true positives ($AUC = 0.7$) of the classifiers. The other classifications lead to AUC scores lower than 0.65: RI ($AUC = 0.62$) is greatly influenced by Sox9. The SE classifier ($AUC = 0.64$) is the only one mostly controlled by CREB. The SY classifier ($AUC = 0.5$) is mostly influenced by a set of TF that includes Sox9, CREB and FOXO among the most relevant, and contributions of Hif2a, NF-$\kappa$B and Runx2 result largely irrelevant.

Table 1 summarizes the results of the classification of the WOMAC domains data recruited in the OAI initiative after binarizing them with the thresholds found with our data. The average accuracy of the six explored classifiers is 0.9794.

**Table 1:** Accuracy of SVM classification models using the Osteoarthritis Initiative (OAI) WOMAC data as targeted outputs. Before SVM classification, WOMAC OAI results were binarized according to the thresholds found with our cohort. The input features used were selected according to the protocol described in the Supplementary Information section “Selected features for OAI data set classification”, and listed in this same section in Additional Figures A2–A7.

Classifier	Accuracy
Left knee funcionality	0.9968
Right knee funcionality	0.9978
Left knee pain	0.9622
Right knee pain	0.9644
Left knee rigidity	0.9784
Right knee rigidity	0.9773

## Discussion

Knee OA diagnosis is influenced by subjective parameters (i.e., WOMAC domains)^13^, which impacts treatment decisions. To reduce the subjectivity in knee OA diagnosis, the present study aimed to characterize seven clinical knee OA descriptors commonly used by examining the contributions of synovial and chondrocyte-derived information. Specifically, a cohort of 51 women with Kellgren–Lawrence grades 2–3 was classified with SVM. However, logistic regression would be another plausible choice, and yet similar results to the ones provided by the SVM would be obtained, as the SVM and logistic regression loss functions are similar. However, SVM is more robust to outliers and overfitting, thanks to the tunning of parameter C. Accordingly, SVM was used to evaluate the value of biological data to inform objectively about the clinical stratification of patients^39^.

First, we assessed how the descriptors that play a role in the knee OA clinical manifestations can be classified based on a combination of these very same descriptors. Fo Remarkably, pain catastrophism (CA) was the leading input for the classification of the joint pain dimension (JP) of the WOMAC questionnaire (Fig. 1). Pain catastrophizing is characterized by a patient’s tendency to magnify pain stimuli and feel helpless in the context of pain^5^. It has been argued that the assessment of CA in response to a specific stimulus might account for most of the variance in pain reports^14–16^. Accordingly, the influence of CA in our classification of knee OA descriptors suggests that JP is not assessing accurately the pain due to noxious stimulus, highlighting potential bias in current diagnosis systems^17,18^. At the same time, DE was the second leading feature for JP classification. A previous study demonstrated a high correlation between high WOMAC pain scores and depression^19^. Furthermore, knee OA patients were found to have increased negative beliefs and mental health issues^20^. These results might emphasise a bidirectional relation regarding the feeling of pain and mental health: patients who develop depression might be more prone to develop low tolerances to pain (a phenomenon called central sensitization, SE) and reduce their physical activity (i.e., sedentarism or static postures). In other words, our classifications support the idea that pain perception contributes to depression and emotional distress in knee OA patients, which in turn, feedback loops with pain reporting. Accordingly, SE was the leading feature for DE classification, which is consistent with previous findings: in individuals with knee OA, the presence of enlarged pain areas was associated with more persistent and severe pain, as well as higher anxiety levels. These findings were interpreted as a sign of altered central pain processing^21^ Beyond its influence on DE, SE played an important role in classifying the WOMAC domains within our cohort, in general, since it resulted to be one of the leading influential features (Fig. 1A). Accordingly, SE might increase WOMAC scores indicating secondary influences in the outcome of these questionnaires.

FU had also a clear influence on the classification of RI, which matches previous findings by Wolfe et al.^19^ In turn, FU classification was greatly affected by SE and CA. These results suggest that the fear of movement (kinesophobia) might impose circular interactions among cognitive factors and pain perception, as Wong et al.^22^ reported previously for the case of CA^23^. There is a growing consensus that disability symptoms in knee OA patients rest upon various factors, including central pain processing mechanisms^20^. As a result, a person may distance herself from activities and social situations to avoid the appearance of pain, increasing the risk of developing an unhealthy lifestyle and depression^24^. In fact, a previous study predicted that static postures might decrease chondrocyte anabolic activity which decreases the deposition of extracellular matrix^12^.

Figure 1 further suggests that subjective clinical descriptors influence the classification of inflammation (i.e., SY). Although knee pain (JP) has been linked to joint inflammation^25^, our findings, as illustrated in Fig. 1A, do not reflect this behaviour: RI led the way in classifying SY with AUC = 0.86. The lack of mechanistic significance of these inputs highlights the need for new objective biological markers in knee OA clinical-decision making. In this sense, serum and urine biochemical markers (in combination with magnetic resonance imaging) have been used to establish knee OA phenotype classification systems^9^. As the pain might be correlated to the intensity of articular cartilage degradation and a pro-inflammatory environment^26^, there is a growing interest in exploring the synovial fluid inflammatory molecules^10,11,27^. However, to the best of our knowledge, no study has specifically explored the capacity of synovial fluid markers to increase our understanding of existing knee OA diagnosis descriptors. Hence, we explored the potential of measurable molecules in synovial liquid to provide insights into KAO clinical features. Figure 2A reveals a high potential of leptin to explain the classification of knee OA patients according to clinical descriptors. Specifically, the relatively leading role of leptin in the good discrimination of patients regarding the SY levels ($AUC > 0.67$) suggests that the endocrine action of leptin might have an important role in joint inflammation stratification, especially in such joints not affected by over-compression. Leptin is a hormone produced by white adipocytes, and it is overexpressed in individuals with metabolic syndrome. This syndrome is characterized by the presence of systemic low-grade inflammation, that has the potential to induce pathological processes in several tissues. In the specific case of chondrocytes, leptin has been linked with increased production of iNOS (the main producer of NO)^28^. Moreover, leptin shows a synergic effect with IL-1$\beta$ on the production of NO in human articular cartilage chondrocytes^29^. Besides, leptin has been associated with increased production of matrix-degrading enzymes (i.e., MMP1 and MMP3)^30^. Other studies demonstrated that leptin alone can induce the synthesis of PGE2 (a molecule associated to modulate inflammatory pain), IL-6, IL-8, MMP1, MMP3 and MMP13 in cartilage explants^31,32^.

But, the main influencing parameter for SY and JP classification was MCP1 (Fig. 2A). This suggests that MCP1 might be used as a new biomarker to evaluate inflammation as it efficiently classifies SY ($AUC = 0.67$, Fig. 2B). Curiously, a study relates structural progression and pain with the abundance of activated macrophages^33^. For SY classification, VEGF-A emerges as the second most influential parameter, which is a reasonable inference: once macrophages are recruited, they stimulate VEGF production, which increases angiogenesis and synovitis. This creates a positive feedback loop where macrophage recruitment contributes to the perpetuation of joint inflammation, thereby leading to more severe forms of knee OA^34^. Thus, our results suggest that patients with higher levels of MCP-1 might be more prone to have painful forms of knee OA. MCP-1 might be used as an objective factor to differentiate pain induced by sensitization from pain induced by nociception and adjust the clinical decision accordingly.

We used SF information as initial conditions to a previous RNM for articular cartilage chondrocytes to obtain patient-specific information about the TF proteomic profile. Figure 3A illustrates that TF that usually are activated in healthy chondrocytes (i.e., FOXO, Sox9, and Cited2) contribute more in the classification tasks, while the hypertrophic (i.e., Runx2 and Hif2) and acute inflammatory (i.e., NF-$\kappa$B) TFs do not greatly affect the stratification of patients. But, AUC scores in Fig. 3B (with an overall mean of 0.56) suggest that there is not a clear pattern within knee OA patients. This fact might reflect the pleomorphic behaviour of osteoarthritic chondrocytes associated with unstructured activation of intracellular signalling pathways, which leads to a microheterogeneity of cellular reaction patterns within subjects^35^. However, the AUC analysis supports the use of TF for the classification of JP because it discriminates false positives ($AUC = 0.3$). Previous studies with animal models demonstrate that FOXO has protective functions in the response of cartilage to joint trauma and mechanical overload, and is downregulated by pro-inflammatory mediators (i.e., TNF-$\alpha$ and IL-1$\beta$)^36,37^. This and the poor influence of NF-$\kappa$B might help understand why clinical manifestations are insufficiently explained by acute nociceptive pain mechanisms (i.e., tissue damage and acute inflammatory responses). Our results highlight an important role of systemic low-grade inflammation in knee OA (overall leading role of leptin in Fig. 2). However, the relatively low AUC scores point out the need for further corroboration of this hypothesis in larger (longitudinal) cohorts.

Before performing the classification tasks, the clinical descriptors underwent a binarization process with a mean accuracy of 0.83 (see Supplementary Material 1). To validate thresholds, we investigated their application in the baseline OAI dataset. Our findings revealed that the accuracy was above 0.95 for six of the explored binarized outputs. We also examined whether the sensitization threshold matched the threshold recommended by Pujol et al.^38^) and both thresholds were the same mean ($accuracy = 0.67$) on the tests sets.

As with any study, our investigation has limitations: the most notable is the need for confirmation of our presented results in a larger cohort. Besides, according to the size of our data set, we implemented a nested leave-one-out validation. When applying this method, the train set remained highly consistent across each fold. Arguably this particular form of cross-validation may introduce higher variance in the error estimate, yet represents one of the optimal strategies to take most advantage of small data set^39^. Another weak point is the elevated number of features used when SF and TF are explored as inputs, which limits the algorithm to work optimally. Besides, TF data come from a model that was not validated using in-vitro/in-vivo data. Also, we should keep in mind that we used a linear classifier, which cannot model nonlinear relationships in the data. Furthermore, the current study has a cross-sectional design, which difficult to extract conclusions regarding the causality between biomarkers and current knee OA diagnostic systems. However, our findings can guide the design of future studies based on the most influential associations identified.

In conclusion, to the best of our knowledge, this study is the first to explore potential biomarkers that could be related to common diagnostic criteria for knee OA, such as WOMAC scores, pain catastrophism, sensitization, and depression, to potentially increase objectivity in knee OA patient’s stratification. The unique application of Support Vector machine-based classifiers in combination with a regulatory network-based model allows for a more objective understanding of knee OA based on synovial fluid biomarkers. However, it is important to acknowledge that limitations exist, and larger cohorts and longitudinal studies are needed to fully map out objective descriptors for knee OA diagnosis.

## Methods

**Figure 4:**
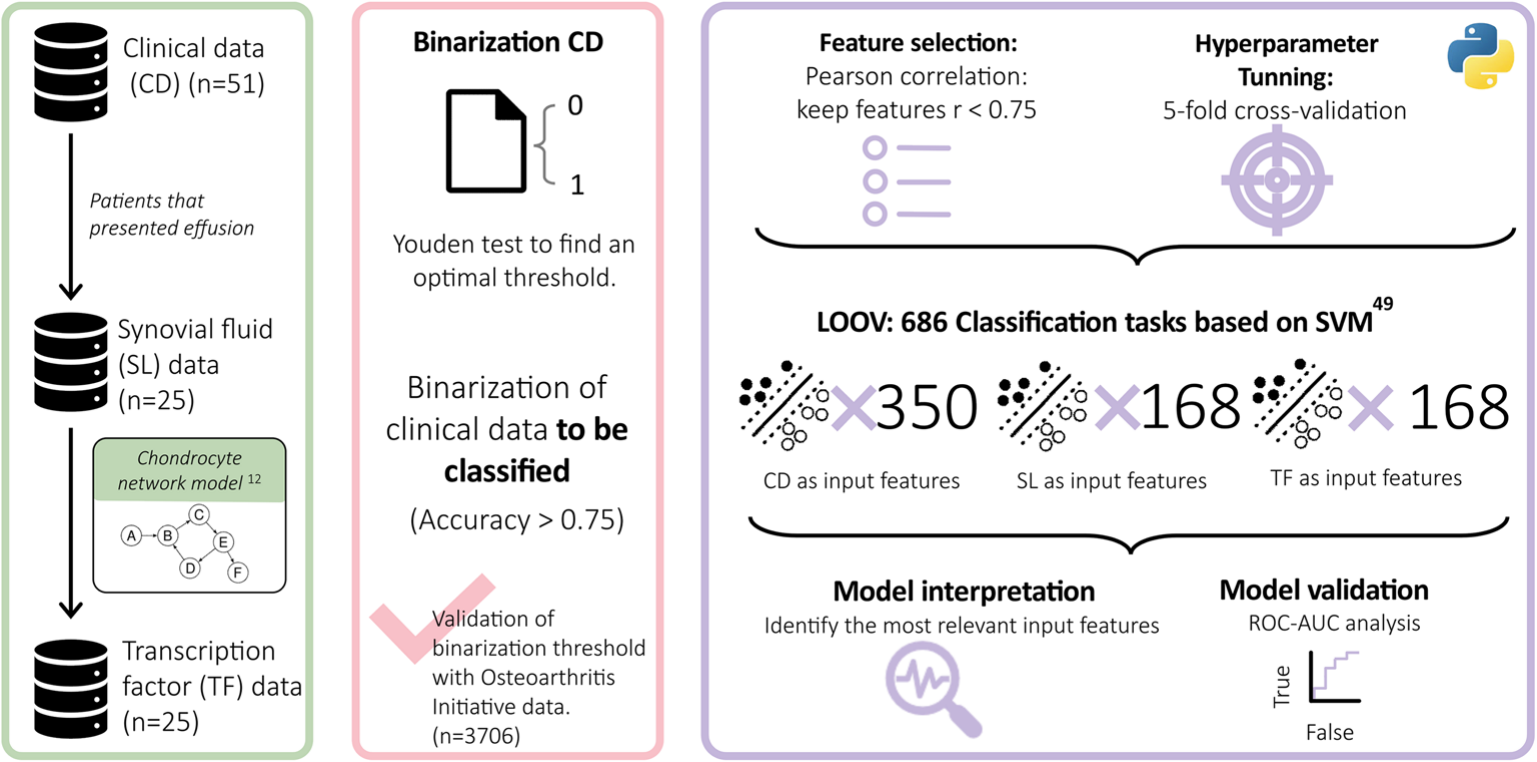
The pipeline of the study: models and data sets (within green boxes); clinical data binarization and its validation (inside the pink box); and support vector machine-based (SVM) classification tasks (within purple boxes) in a nested leave-one-out validation (LOOV).

We explored how to classify subjects in a cohort of women (n = 51) with Kellgren–Lawrence grade 2–3 knee OA, described extensively in terms of inclusion and exclusion criteria by Tassani et al.^40^ From those patients that presented effusion (n = 25) synovial liquid (SL) extraction was performed and measured through Luminex$\circledR$ to obtain information related to inflammatory soluble factors. Such inflammatory mediators were further used as input data for a qualitative dynamical system of a regulatory-network based model (RNM) that summarizes chondrocyte mechanobiological and intracellular activity^12^. From the RNM we obtained personalized virtual information regarding the activation level of seven transcription factors (TF). An overview of the methodoly followed is depicted in Fig. 4.

But, the primary contribution of this manuscript is the characterization of patients regarding the level of seven clinical descriptors (catastrophism (CA), depression (DE), functionality (FU), joint pain (JP), rigidity (RI), sensitization (SE), and synovitis (SY)), which were binarized for the classification purposes with a Youden test. Three sets of data were used as input for the SVM-based classifiers: (i) the most appropriate set of knee OA descriptors; (ii) SL inflammatory data (n = 25), and (iii) in-sillico TF activation levels. All in all, lead to the exploration of 21 classification tasks. The binary classification tasks were validated using the receiver operating characteristic curve (ROC-AUC) analysis. The binarization thresholds were further validated in the baseline of the osteoarthritis initiative (OAI) data set.

### Cohort description

At the Rheumatology/Orthopaedic Surgery Department of the Hospital del Mar (Barcelona, Spain), a cohort was built revising the clinical history of patients diagnosed with knee OA. The study followed the Good Clinical Practice guidelines and the Declaration of Helsinki, and the Clinical Research Ethical Committee approved the protocol (2016/6747/I). All participants signed an approved informed consent and agreed to do a wash-up treatment for 3 months of intra-articular hyaluronic acid infiltrations, 1 month for oral or intra-articular corticoids, and 1 week of nonsteroidal anti-inflammatory or opioid drugs. A total of 51 women were selected who presented radiographic signs of moderate knee OA (Kellgren–Lawrence grade of 2/3) and symptomatology (pain, dysfunction and/or effusion) in the last 3 months. Patients who presented any sign of secondary OA were excluded (meniscectomy, inflammatory or connective tissue diseases, overuse of the joint). Women with effusion (n = 25) were eligible for synovial liquid extraction^40^.

### Description of the output labels

The targeted OA descriptors used for the stratification of patients were:

- Joint Pain (JP): it is assessed using the WOMAC pain scale, where patients rate the level pain experienced during five quotidian (i.e., walking, sitting or standing) activities. For each of these activities, the level of pain felt is reported on a 5-point Likert scale ranging from “none” to “extreme”^41^.
- The functionality of the joint (FU): it contains the results of the WOMAC functional impairment questionnaire that summarizes 17 items with 1—5 Linkert scale responses.
- The rigidity of the joint (RI): it is the WOMAC stiffness subtest contains two categories and is used to rank the rigidity of the joint.
- Central pain sensitization or pain hypersensitivity (SE): Defined by the International Association for the Study of Pain as “increased responsiveness of nociceptive neurons in the central nervous system to either normal or subthreshold afferent input”^42^. SE was evaluated following the protocol described in Pujol et al.^38^
- Effusion (EF): it is a radiographic factor (echography), post-analysed and graded by an expert^38^. It is a measure of the liquid accumulated inside the synovial joint.
- Depression and anxiety (DE): It is obtained from the Hospital Anxiety and Depression Scale (HAD), which measures core symptoms of anxiety and depression without including physical symptoms^43^.
- Catastrophism (CA): based on the Pain Catastrophism Scale, it assesses catastrophic thinking related to pain with or without chronic pain. CA refers to the tendency to focus on, and magnify, ache sensations, and to feel helpless in front of pain^16^.

Originally, these descriptors were collected in a discrete non-binary form, but when used as targets to predict, we converted them into binary categorical variables (i.e. targeted labels). This was done by selecting a threshold that best divided the cohort into two groups. The optimal threshold for each variable was determined through a grid search (see Supplementary Material 1) to maximise the Youden’s index:

1 $$\begin{aligned} J=\frac{\text {TP}}{\text {TP} + \text {FN}}+\frac{\text {TN}}{\text {TN} + \text {FP}}-1 \end{aligned}$$

*TP* refers to true positives, *FN* refers to false negative, *TN* refers to true negative, and *FP* refers to false positive. Maximizing Youden’s index was equivalent to achieving the highest value of the sum of sensitivity (first term on the right side of the equation) and specificity (second term). However, if the accuracy across the entire fold of the leave-one-out validation was less than 0.75 in the train set, we omitted such descriptor as an output label because we assumed that it could not be binarized properly.

### Input features selection

Three types of input features were used for the classification of 7 clinical OA descriptors. A total of 21 classification tasks were explored. The first set of input features was the continuous form of the same clinical OA characteristics. For this case, feature selection was done ahead of binarization, based on the numerical values of the input and output variables. Hence, both Pearson and Spearman are good options. Yet, the Pearson correlation test was preferred because it measures linear relationships among variables, according to the linearity of the kernel of the SVM. We aimed to reduce any possible redundancy of descriptors by avoiding high linear dependency relationships among the input and output sets. Specifically, we excluded those cases with Pearson correlation coefficient $(r) > 0.7$ (see Fig. A1). This ensured that the identified features were completely independent and had no strong linear relationship with the targeted output. To this end, a correlation matrix using Python 7.29.0 (corr. method from pandas library) was generated, which helped identify the highly linearly correlated features regarding the output label.

Synovial fluid (SF) samples from 25 patients were obtained through aspiration and analysed with Luminex$\circledR$ technology to measure the amounts of:

- pro-inflammatory cytokines: Interleukin 6 (IL-6), Interleukin 8 (IL-8), Interleukin 4 (IL-4), Tumor necrosis factor alpha (TNF-$\alpha$), Interleukin 18 (IL-18), Interferon-gamma (INF-$\gamma$), Interleukin 17 (IL-17). These molecules contribute to the degeneration of the articular cartilage and inflammation^44^.
- Interleukin-1 receptor antagonist protein (IL-1RA) inhibits the activity of the Interleukin-1$\beta$ (IL-1$\beta$) receptor.
- Monocyte chemoattractant protein 1 (MCP1) recruits monocytes to inflammation sites produced by tissue injury, propagating inflammation and tissue damage^45^.
- Vascular endothelial growth factor A (VEGF-A) increases angiogenesis^46^.
- Leptin modulates the inflammatory processes and articular cartilage remodelling^47^.

**Figure 5:**
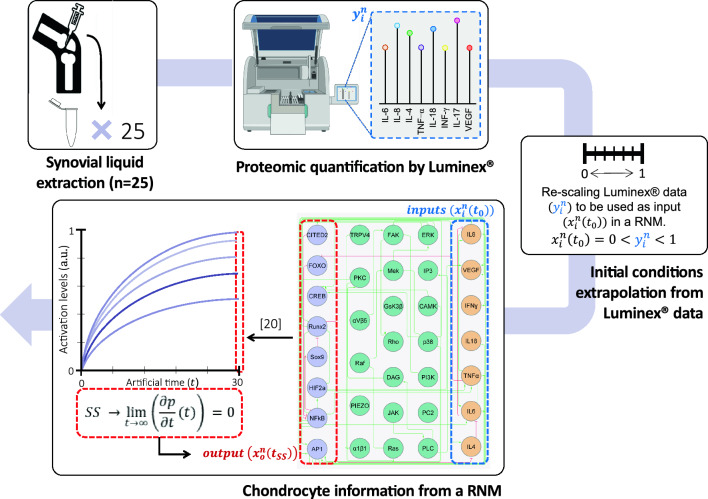
Overview of the methodology followed for obtaining patient-specific virtual information about the activation of transcription factors. (**A**) Synovial liquid from patients who presented effusion was extracted. (**B**) Luminex technology was used to measure soluble inflammatory mediators $y_i^n$, $i =$ IL-8, IL-6, IL-4, TNF-$\alpha$, IL-18, IFN-$\gamma$, IL-17, VEGF. (**C**) Such information is re-scaled between 0 and 1 to be used as an input ($x_i^n(t_0)$) in a regulatory network-based model of a chondrocyte^12^. (**D**) The model converts a static graph (a simplified version of the original published at^12^) into a system of ordinary differential equations following Mendoza et al. methodology^48^. 25 simulations were run with Segarra-Queralt et al.^12^ to obtain patient-specific information about the activation of transcription factors ($x_o^n(t_{SS})$, $o =$ CITED2, CREB, Runx2, FOXO, SOX9, HIF2a, NF-$\kappa$B, AP1).

AC is nourished through the SF. Hence, diffusion of the aforementioned molecules to articular cartilage influences the chondrocyte’s metabolism. Thus, the transcription factor proteomic profile might provide valuable data to characterize patients biologically. Accordingly, and because articular cartilage samples cannot be collected in the patients without causing any serious damage, information from a chondrocyte RNM was used^12^. The RNM models the concentration of molecules (i.e., proteins) by time-dependent variables. The synthesis of each network node (i) is regulated by rate equations in the form $\frac{\textrm{d}x_i}{\textrm{d}t}=f_i(x_n)$, which depends on regulation nodes (*n*). The RNM can capture the intracellular channelling of external signals into identifiable cellular behaviours^48^. In this work, we have used the patient SF as an external signal of a chondrocyte RNM previously developedSegarra23. From the simulations, we generated personalized synthetic information about 8 TF: Activator Protein 1 (AP1); cAMP response element-binding protein (CREB); forkhead box 1 (FOXO); Nuclear factor kappa-light-chain-enhancer (NF-$\kappa$B); transcription factor Sox9 (Sox9); Cbp/p300-interacting transactivator 2 (CITED2); Runt-related transcription factor 2 (Runx2); Hypoxia-inducible factor 2-alpha (HIF2a). Detailed integration can be found in Additional Information and summarized in Fig. 5.

### Mathematical formulation

SVM is a supervised approach for classifying^49^. It divides the samples into two groups by identifying the separating hyper-plane with the largest distance (margin) to the nearest training data points, called the support vectors. In this work, a binary linear SVM was used to predict a class $y_n\in [0,1]$, based on the input training set $x_n\in \mathbb {R}^M,n=1,\ldots ,N,$ where *M* was the number of features and *N* the number of samples. A linear hyperplane (a line if m = 2 or a plane if m = 3) can be written as the set of points x satisfying

2 $$\begin{aligned} \omega ^Tx-b=0 \end{aligned}$$

where $b\in \mathbb {R}$ was the ordinate at the origin and $\omega \in \mathbb {R}^M$ was the normal vector to the hyperplane. The algorithm found a prediction function that categorized most samples correctly by minimizing:

3 $$\begin{aligned} min_{\omega ,b,\zeta } \frac{1}{2}\omega ^T\omega + C\sum _{n=1}^N{\zeta _n} \end{aligned}$$

subject to

4 $$\begin{aligned} y_n(\omega ^T(x_n) + b) \ge 1-\zeta _n, \zeta _n\ge 0, n=1,...,N \end{aligned}$$

$y_n$ was the known label corresponding to the sample $x_n$, $\zeta _n$ is the extent to which the margin constraint on $x_n$ can be violated, and *C* was the penalty term that controlled the number and severity of the violations to the margin. Given a sample *x*, the algorithm classified it by determining on which side of the hyperplane was $y_n$ (i.e., whether $\omega ^T(x_n)+b$ was positive or negative.

To ensure the maximum performance of the classifier, hyperparameter tuning is done before the training step based on k-fold cross-validation ($k\ =\ 5$). Once the best hyperparameters were found, the SVM was built in Python (Version 3.9.7)^50^. Furthermore, according to the size of the data set, leave-one-out validation was applied. Thus, we have trained $n=50$ models when clinical data is used as input feature, and 24 when synovial fluid and transcription data are used as inputs. With the linear SVM, the size of each weight $\omega _m\in \mathbb {R}$ relative to the other ones indicated how important the m-th feature was for the separation. With the leave-one-out validation, the $\bar{\omega }_m=\frac{\sum ^{n-1}\omega _m}{n-1}$ was obtained. The $\bar{\omega }$ value was normalized between 0 and 1 for each *M* of the $x_n$ for visualization purposes. Finally, the performance of each classifier was evaluated using a nested receiver operating characteristic curve (ROC-AUC) analysis, and mean AUC scores were reported^50^.

### Thresholds validation

We validated the binarization of features in the Osteoarthritis Initiative (OAI) baseline dataset using the thresholds found for the WOMAC domains (see Table A1). The OAI dataset recruited 4796 individuals in the United States. To minimize the noise in the data, we excluded individuals who had either knee history of knee surgery (i.e., arthroscopy, ligament repair, meniscectomy) and participants with less than 80% of variables completed, resulting in 3706 subjects. Pearson correlation was used for dimensionality reduction, to remove highly correlated variables ($r>0.7$), leaving 785 features for analysis. Finally, information gain was used to identify the first 50 most important features (see Figs. A2, A3, A4, A5, A6 and A7). Information gain calculation was based on the reduction in entropy of the dataset to evaluate each feature’s importance^51^. Hyperparameter tuning was then performed, followed by SVM classification for six targeted outputs: WOMAC pain, functionality and rigidity of the right and left knee. A detailed description of this process can be found in Additional Information.

Topical subheadings are allowed. Authors must ensure that their Methods section includes adequate experimental and characterization data necessary for others in the field to reproduce their work.

### Supplementary Information

Supplementary Information.

## Data Availability

The data that support the findings of this study are available from the Hospital del Mar but restrictions apply to the availability of these data, which were used under the terms of the informed consent signed by the patients for the current project, and so are not publicly available. Data are however available from the corresponding author, Jérôme Noailly, upon reasonable request and with permission of the Ethical Committee of the Hospital del Mar. Data for the thresholds validation is available at https://nda.nih.gov/data_structure.html?short_name=clinoq01OAI web-page.
