# Supplementary material for: Characterization of clinical data for patient stratification in moderate osteoarthritis with support vector machines, regulatory network models, and verification against osteoarthritis Initiative data
Source: Sci Rep. 2024 May 23;14:11797. doi: 10.1038/s41598-024-62212-x (PMC11116450; doi:10.1038/s41598-024-62212-x)
Supplement: Supplementary file 1 — Supplementary Information. [file 41598_2024_62212_MOESM1_ESM.pdf]

Supplementary Information

Feature Correlation

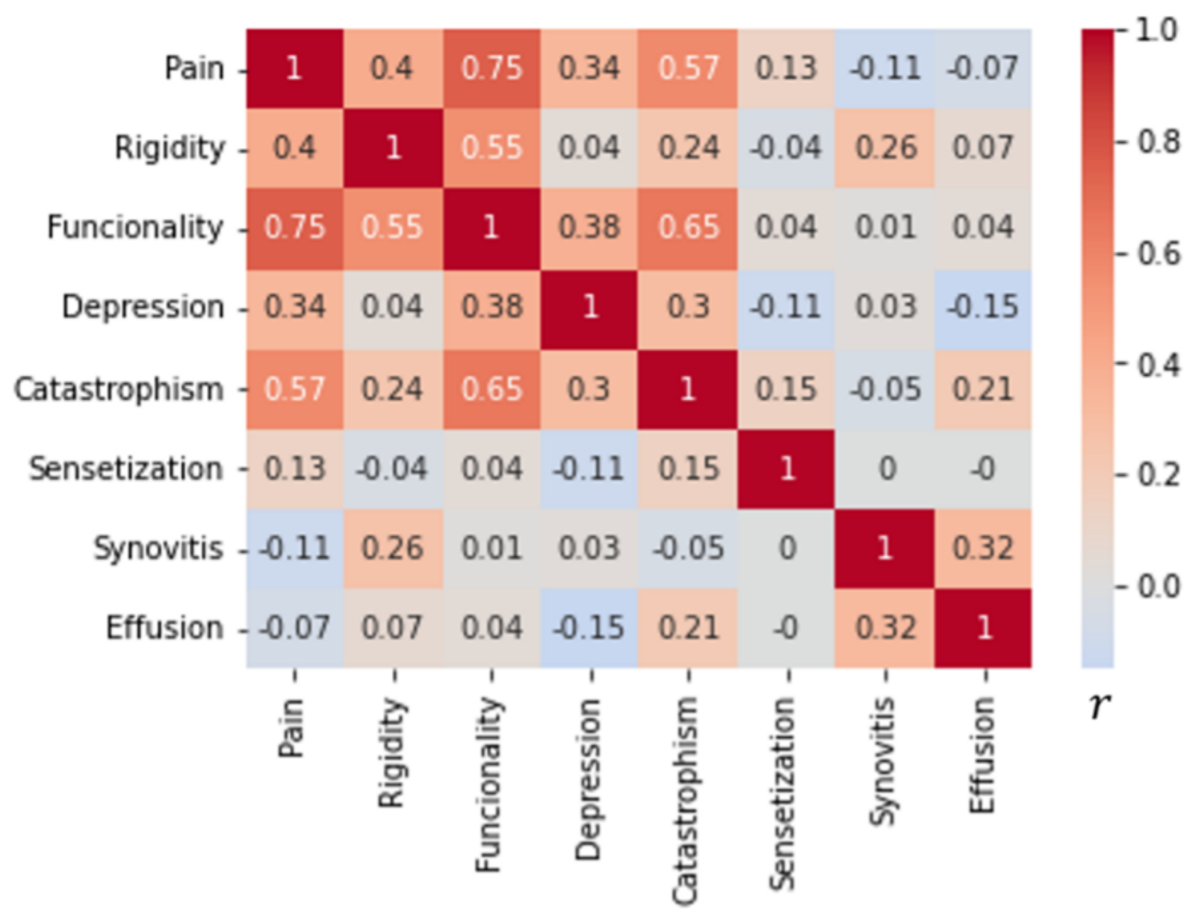

Additional Figure A1. Heat map of Pearson's r, which represents the pairwise correlation between knee osteoarthritis descriptors.

Threshold exploration

The threshold for 7 out of the 8 classifiers studied is explored to find the best discriminative limit among them. The only classifier that does not require an insightful study is the SE classifier, as there is a clear consensus regarding the limit that excludes patients in two groups.[38] In Table A1 can be found the thresholds used for each classifier in this study with their accuracy on the test set across the entire leave-one-out folds.

Selected features for OAI data set classification

We check whether the thresholds defined for JP, RI, and FU can be extrapolated in another data set, The OAI data set has been used as a validation dataset for the thresholds defined with the HOLOA cohort. But, the OAI dataset contains a lot of information which have to be refined. The information gain[51] was used as a measure for selecting the fifty more influencing features of the OAI cohort. We used the mutual info classify method from the sklearn.feature selection library in Python 3.9.7. You can see in fig A2, A3, A4, A5, A6 and A7 the features used in the classification tasks. The names of the features are related to a specific clinical feature that can be found in the StringOAI web-page.

Classifier	Best threshold	Accuracy
JP	10	0.94
RI	6	0.78
FU	52	0.75
SE	1[38]	-
DE	8	0.86
CA	16	0.88
SY	4	0.75
EF	1	0.73

Additional Table A1. Summary of the thresholds used in the SVM classification tasks for the binarization approach.

	Mean accuracy across LOOV in the test sets		
Classifier	Clinical Data	Synovial Fluid	Transcription factors
JP	0,90	0,83	0,83
RI	0,73	0,70	0,57
FU	0,67	0,43	0,58
SE	0,67	0,74	0,74
DE	0,73	0,91	0,91
CA	0,78	0,70	0,61
SY	0,65	0,87	0,87

Additional Table A2. Mean accuracy of the models across the leave-one-out validation (LOOV) for the three sets of input features.

Obtention of transcription factor data with a Regulatory network model

AC is an avascular tissue nourished through the synovial liquid. Hence, diffusion of the aforementioned pro-inflammatory molecules from synovial liquid to AC is expected to influence the chondrocyte's metabolism. Thus, transcription factor proteomic profile might provide valuable data to characterize biologically KOA patients. Accordingly, and because AC samples cannot be collected in the patients without causing any serious damage, intracellular information from a chondrocyte RNM[12] was used. Specifically, the RNM is a mathematical approach based on ordinary differential equations (ODE) that models the metabolic network of an articular chondrocyte. An ODE RNM models the concentration of molecules (i.e., proteins) by time-dependent variables. The synthesis of each network component (i), or node, is done by rate equations in the form $(dx_i)/dt = f_i(x_n)$ expressing the production of each component of the system as a function of the concentration of the regulation nodes (n). Modelling the molecular networks as dynamical systems can capture the intracellular chaneling of externals signals into coherent and clearly identifiable cellular behaviours[48].

In this work, we have used the patient SF profile to stimulate as an external signal a chondrocyte dynamic RNM previouSFy developed.(Segarra-Queralt et al., 2023) From the simulations, we generated personalized synthetic information about 8 transcription factors: Activator Protein 1 (AP1); cAMP response element-binding protein (CREB); forkhead box 1 (FOXO); Nuclear factor kappa-light-chain-enhancer (NF-κB); transcription factor Sox9 (Sox9); Cbp/p300-interacting transactivator 2 (CITED2); Runt-related transcription factor 2 (Runx2); Hypoxia-inducible factor 2-alpha (HIF2a). The methology is sumarized in fig 5.

Synovial liquid acquisition

Synovial fluid was acquired from patients who presented effusion, following the standard protocol of the Luminex® assay.

Data normalization

The synovial fluid data obtained from Luminex® was normalized. Each molecule's values were ranked based on a calibration curve (e.g., VEGF-A ranged from 10 to 20, TNF-α from 35 to 90). As the RNM model by[12] can only integrate external inputs from 0 to 1, we rescaled the values for each patient between 0 and 1, using the calibration curve as a reference. The maximum value of the calibration curve was set to 1, and the minimum value to 0. Then, the synovial liquid value was re-scaled accordingly.

Running simulations

For each patient (n=25), 8 synovial fluid molecules were used as initial conditions in the RNM developed by[12]. From this model, we obtained the relative activation profile of 8 transcription factors, also ranging from 0 to 1.

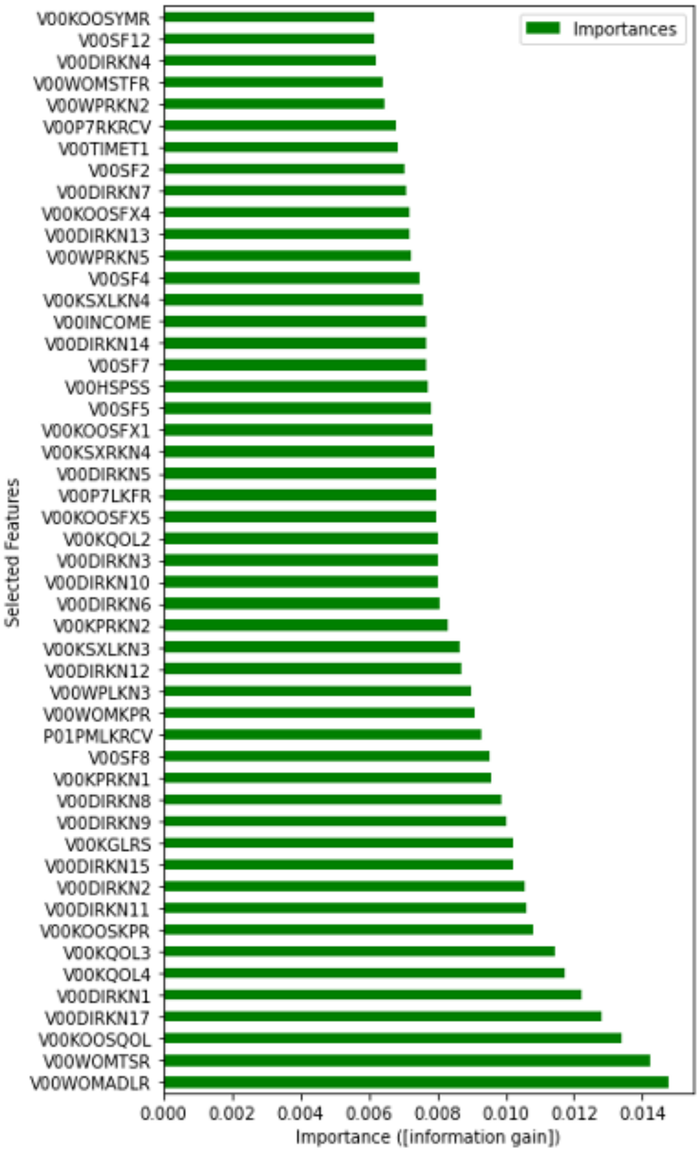

Additional Figure A2. Input features used for the classification of WOMAC disability domain of the left knee.

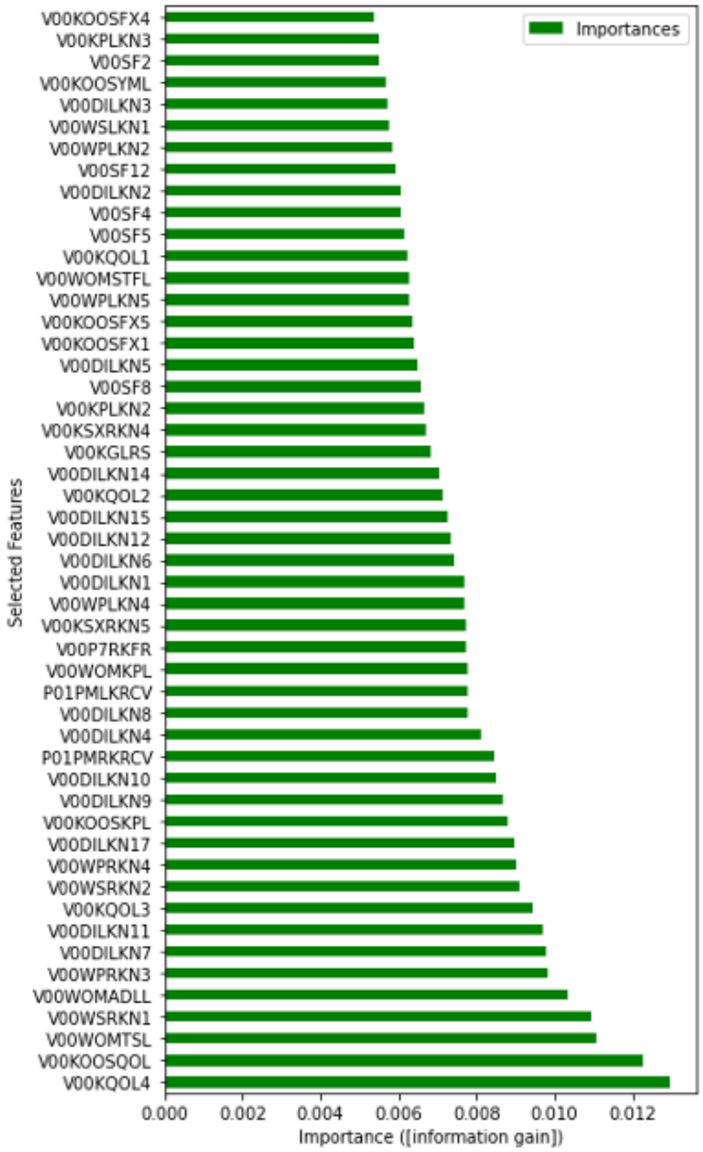

Additional Figure A3. Input features used for the classification of WOMAC disability domain of the right knee.

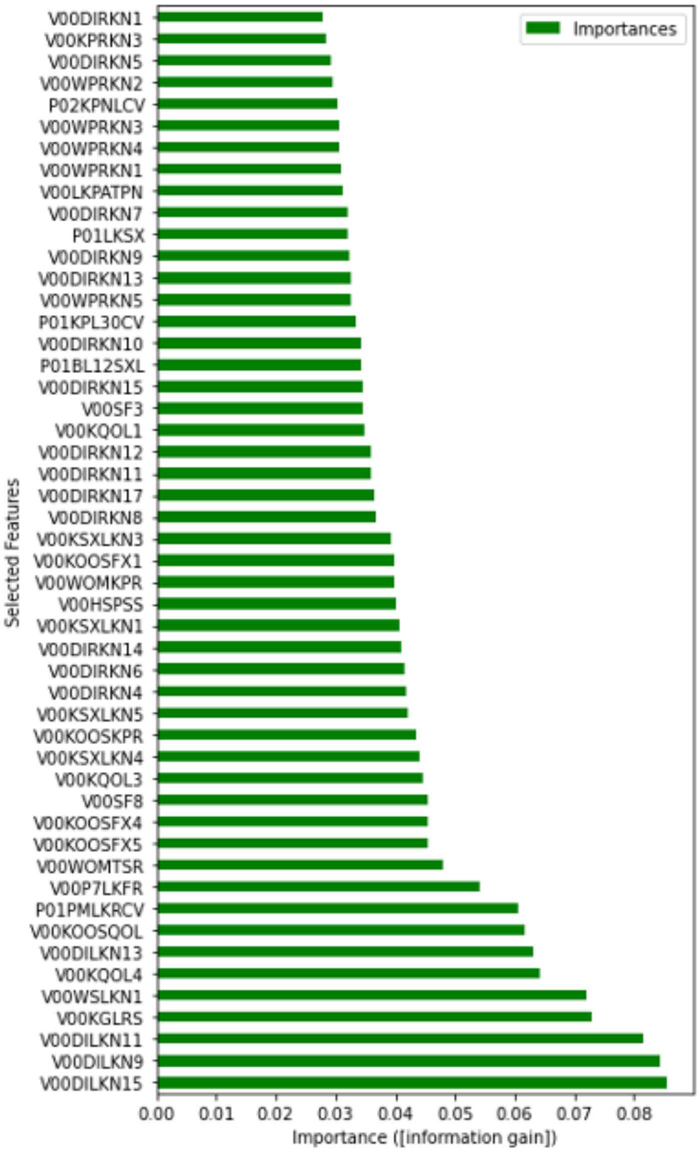

Additional Figure A4. Input features used for the classification of WOMAC rigidity domain of the left knee.

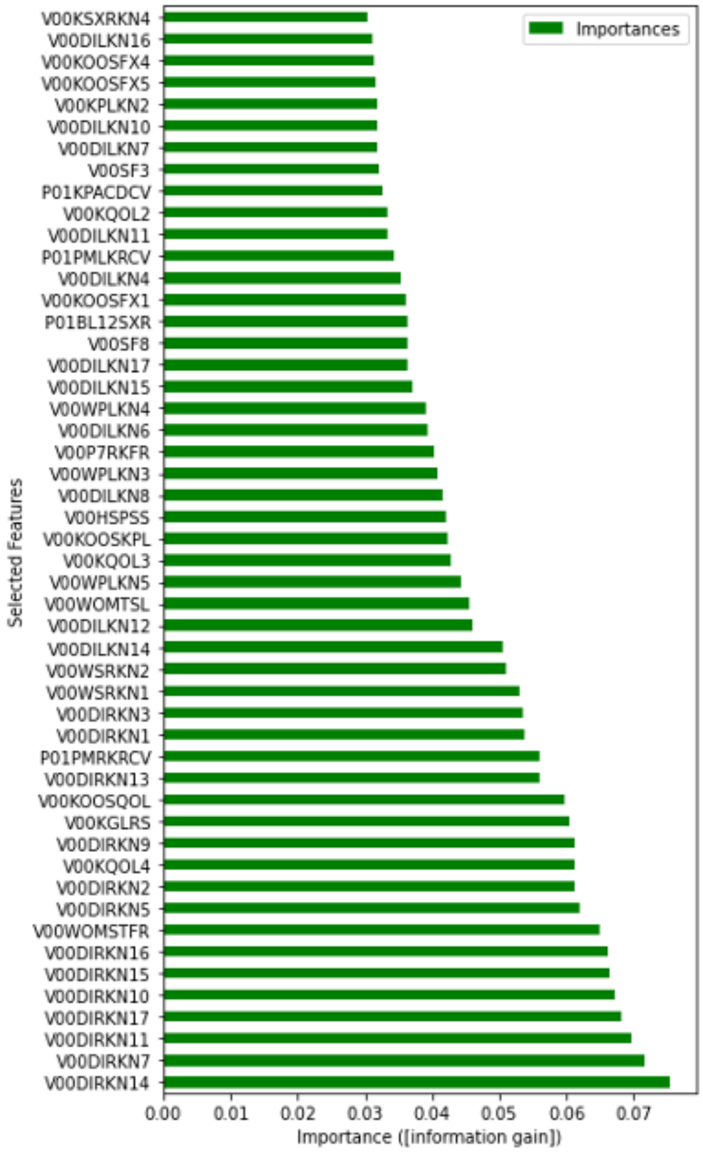

Additional Figure A5. Input features used for the classification of WOMAC disability domain of the left knee.

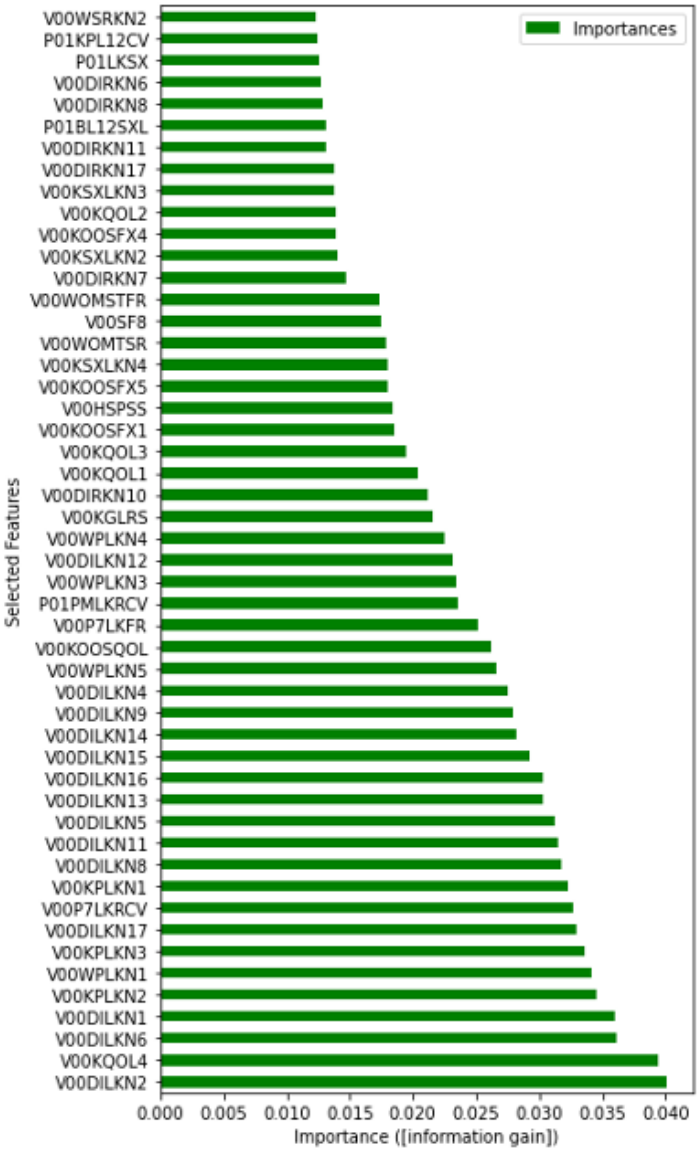

Additional Figure A6. Input features used for the classification of WOMAC rigidity domain of the right knee.

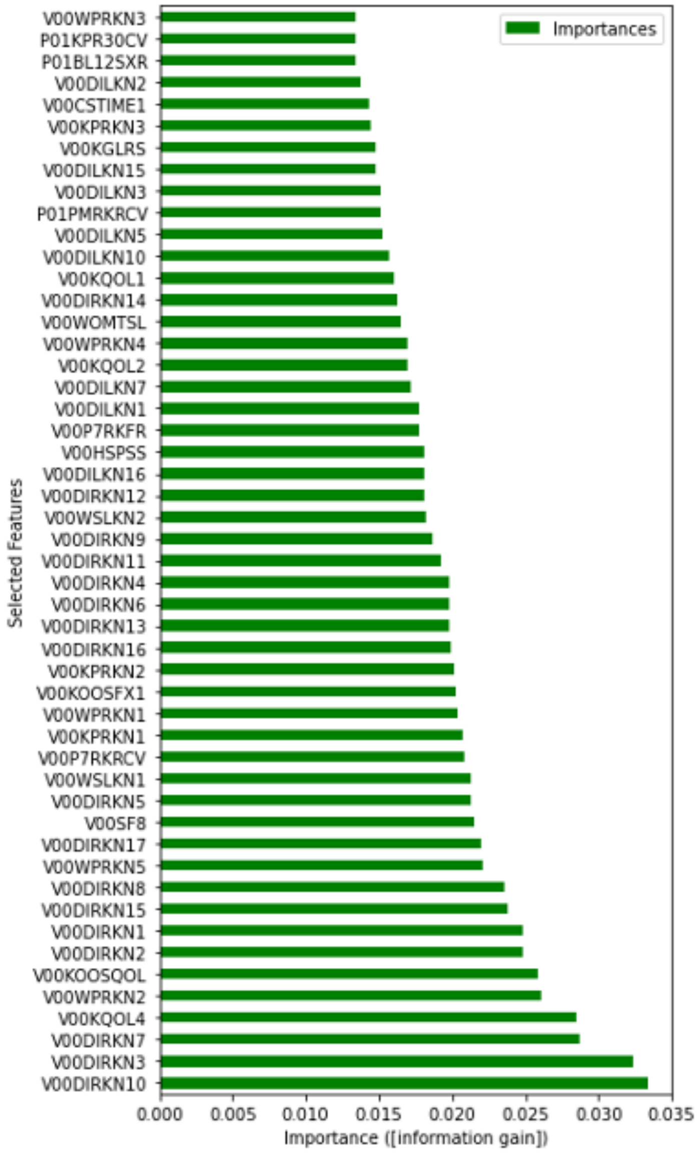

Additional Figure A7. Input features used for the classification of WOMAC pain domain of the left knee.
